# Application of Artificial Intelligence in the Endoscopic Diagnosis of Gastric Cancer and Precancerous Lesions

**DOI:** 10.3390/diagnostics16142196

**Published:** 2026-07-14

**Authors:** Mengmeng Su, Siyang Fu, Wanying Liao, Aiming Yang

**Affiliations:** Department of Gastroenterology, Peking Union Medical College Hospital, Chinese Academy of Medical Sciences, Peking Union Medical College, Beijing 100006, China

**Keywords:** artificial intelligence, endoscopy, gastric cancer, precancerous lesions, deep learning

## Abstract

Gastric cancer is a globally prevalent malignancy, with early detection being pivotal for improving patient survival. While endoscopy remains the diagnostic gold standard, it frequently faces challenges such as missed lesions and operator dependency. Artificial intelligence (AI) has emerged as a powerful tool to address these limitations. This narrative review synthesizes recent evidence from PubMed and Web of Science, focusing on four core functional domains of AI-assisted gastric endoscopy: lesion detection and characterization, margin delineation, invasion depth prediction, and blind-spot monitoring. Furthermore, we summarize current limitations, including single-center data biases and algorithmic “black-box” issues, and discuss future directions such as multimodal data integration and real-time video analysis systems. Ultimately, carefully validated AI represents a vital clinical adjunct that holds great potential to significantly enhance diagnostic accuracy and patient outcomes.

## 1. Introduction

Gastric cancer ranks as the fifth most commonly diagnosed malignancy and the third leading cause of cancer-related mortality globally, with over 1 million new cases and 769,100 deaths reported in 2022 [1]. East Asia, particularly China, bears the heaviest burden worldwide [2]. The prognosis for gastric cancer patients is closely linked to the stage at diagnosis. The five-year survival rate for advanced gastric cancer is less than 30%, whereas that for early-stage disease exceeds 90% following endoscopic resection [3,4]. Thus, the early identification of gastric cancer and its precancerous lesions (e.g., atrophic gastritis, intestinal metaplasia) is indispensable for interrupting malignant progression and optimizing patient outcomes [5,6].

Endoscopy plays a vital role in the diagnosis, surveillance, and treatment of gastric cancer and its precancerous lesions. The standard diagnostic workflow typically begins with white-light imaging (WLI) screening to localize macroscopic abnormalities in the gastric mucosa. Subsequently, image-enhanced endoscopy (e.g., narrow-band imaging [NBI], blue-light imaging [BLI], or linked color imaging [LCI]) combined with magnifying endoscopy (ME) is employed to assess microvascular and microsurface patterns for the qualitative diagnosis of suspicious lesions. Conventional chromoendoscopy (CE) or endoscopic ultrasonography (EUS) may also be utilized when necessary to delineate lesion margins and assess invasion depth [7,8].

Despite the availability of multiple endoscopic tools, diagnostic accuracy remains suboptimal. The overall missed detection rate for early gastric cancer using white-light endoscopy ranges from 20% to 40% [9,10]. Diagnostic accuracy is particularly limited for specific types such as flat lesions, diffuse-type gastric cancer, and early gastric cancer arising from post-eradication gastritis. Furthermore, diagnostic performance is influenced by operator expertise and visual fatigue. Addressing these challenges to enhance the diagnostic accuracy of gastric cancer and precancerous lesions remains a critical priority in clinical practice.

**Figure 1:**
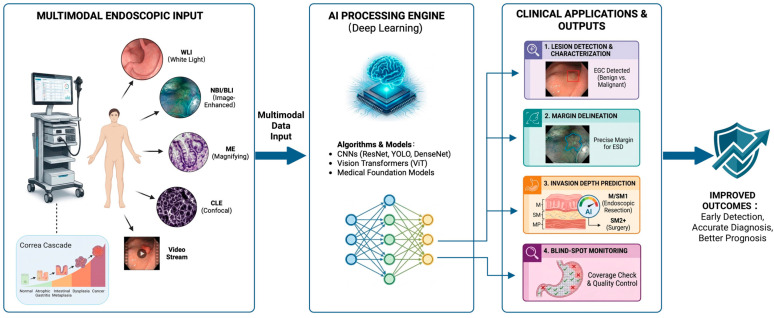
**Conceptual overview of AI-assisted endoscopy for the diagnosis of early gastric cancer and precancerous lesions.** The workflow highlights the transition from multimodal endoscopic data acquisition (**left**) to advanced AI processing engines (**center**), which power four major clinical applications (**right**). Ultimately, this human-AI collaborative loop leads to improved clinical decision-making and patient prognosis. Abbreviations: WLI, white-light imaging; NBI, narrow-band imaging; BLI, blue-light imaging; ME, magnifying endoscopy; CLE, confocal laser endomicroscopy; CNN, convolutional neural network; ViT, vision transformer; EGC, early gastric cancer; ESD, endoscopic submucosal dissection; M, mucosa; SM, submucosa; MP, muscularis propria.

Advancements in artificial intelligence (AI) offer promising solutions to these challenges. Significant breakthroughs have been achieved in research combining AI with endoscopy to enhance the efficiency and accuracy of diagnosing gastric cancer and its precancerous lesions (Figure 1). This review aims to summarize current applications of AI-assisted endoscopy in detecting early gastric cancer and its precancerous lesions, while analyzing existing limitations and outlining future directions for clinical implementation.

## 2. Deep Learning-Assisted Lesion Detection and Characterization

### 2.1. WLI (Static Models)

White light endoscopy (WLE) constitutes the most fundamental endoscopic procedure and serves as a vital tool for monitoring gastric cancer and its precancerous lesions. In recent years, numerous studies have focused on developing artificial intelligence (AI) models for WLE based on deep learning (Table 1). Zhang et al. [11] developed a convolutional neural network model (CNN-CAG) based on the DenseNet121 architecture for diagnosing chronic atrophic gastritis using WLE images. Utilizing a dataset of 5470 gastric antral endoscopic images (70% for training, 30% for testing), the model achieved a diagnostic sensitivity of 0.945 and a specificity of 0.940. It demonstrated detection rates of 95–99% for moderate to severe atrophic gastritis, outperforming expert diagnosis. This model can precisely identify high-risk populations for gastric cancer, facilitating targeted clinical interventions. Yuan et al. [12] developed a multi-classification AI system based on the YOLO model for diagnosing gastric lesions under white-light endoscopy. Incorporating 2980 training images and 1579 validation images, the system achieved an overall accuracy of 93.5% in detecting early gastric cancer. This performance matched that of senior endoscopists and significantly surpassed that of junior practitioners. With AI assistance, the diagnostic accuracy of senior and junior endoscopists for early gastric cancer improved to 94.9% and 94.1%, respectively, with a slight reduction in diagnostic time. This highlights the potential of artificial intelligence as a valuable auxiliary diagnostic tool for endoscopists. Luo et al. [13] developed the Gastrointestinal Artificial Intelligence Diagnostic System (GRAIDS), which was trained and validated using 1,036,496 white-light endoscopy images from six Chinese hospitals of varying tiers. In multicenter testing, its diagnostic accuracy for gastric cancer reached 0.955, with a sensitivity comparable to endoscopy specialists (0.942 vs. 0.945) and significantly superior to that of non-specialist physicians. Notably, a cloud platform was established to support real-time assistance and post-operative review, thereby enhancing diagnostic capabilities in primary care hospitals. Dong et al. [14] further developed the ENDOANGEL-ED system, pioneering the integration of domain knowledge and interpretability into early gastric cancer diagnosis under white-light endoscopy. By extracting and weighting 13 medical features, it achieves “visualization of diagnostic outcomes and rationale,” addressing the traditional “black box” issue. Through multi-center training and validation, it maintains high diagnostic efficacy while significantly enhancing physician trust (4.42 vs. 3.74) and acceptance (4.52 vs. 4.00). Following AI assistance, physician accuracy rose from 70.61% to 79.63%, making this system highly suitable for integration into routine clinical practice.

### 2.2. NBI and ME-NBI Modality

NBI employs green/blue wavelengths to substantially enhance visualization of mucosal surface structures and capillary patterns [15,16]. Studies indicate its diagnostic efficacy surpasses that of white-light endoscopy in detecting early gastric cancer and precancerous lesions [17,18]. However, proficient application of NBI for early gastric cancer diagnosis with favorable outcomes demands extensive expertise and skill. Artificial intelligence offers potential solutions to these challenges. Tang et al. [19] developed an AI model for diagnosing early gastric cancer (EGC) during narrow-band imaging (NBI) endoscopy, utilizing a deep convolutional neural network (DCNN) based on the YOLOv3 framework. The study collected 21,785 NBI images and 20 video clips for model training and validation. The AI model was compared against the diagnostic performance of seven endoscopists (three senior, four junior) to evaluate its auxiliary role for clinicians. Results demonstrated that the AI model’s diagnostic performance (93.2% accuracy) surpassed that of both senior (85.9%) and junior endoscopists (79.5%). It significantly improved diagnostic accuracy for both groups (senior to 89.4%, junior to 84.9%) while achieving 100% EGC detection in the video validation set. To address NBI accessibility limitations, Lei et al. [20] employed a stable diffusion AI model to convert white light endoscopy (WLE) images into virtual NBI (Vir-NBI) images. The model was fine-tuned using 273 authentic NBI images to transform 111 WLE images into Vir-NBI. Assessed by physicians of varying experience levels, Vir-NBI demonstrated diagnostic efficacy comparable to genuine NBI for early gastric cancer. It significantly outperformed WLE in both lesion area consistency rate (43.85%) and overall lesion detection rate (45.33%) versus WLE’s 39.32% and 32.87%, respectively.

### 2.3. CLE and pCLE Models

Confocal laser endoscopy (CLE) is a high-resolution imaging technique enabling real-time in vivo histological observation of gastrointestinal mucosa at the cellular level, thereby achieving “optical biopsy” [21,22]. Studies have demonstrated its diagnostic sensitivity for early gastric cancer and precancerous lesions reaches 98.33%, which is comparable to conventional histopathology [23]. Cho et al. [24] developed an AI-based real-time gastric cancer tissue assessment model utilizing a confocal laser endoscopic system (CLES), trained on 7480 tumor and 12,928 normal tissue images from 43 patients, achieving an AUROC of 1.000 in internal validation. External validation demonstrated diagnostic accuracy of 0.990 and sensitivity of 1.000, outperforming four certified pathologists. AI assistance significantly enhanced pathologists’ diagnostic accuracy and inter-observer agreement, offering potential as an auxiliary tool for pathologists. Liu et al. [25] developed a deep learning-based pCLE computer-aided diagnosis system (CCADS). The model was constructed and trained using retrospectively collected data from 5771 gastric pCLE examinations (comprising 47,462 images and 461 video clips). Offline validation (11,439 images and 667 videos) yielded an accuracy ranging from 95.05% to 99.61% for images and 92.80% to 98.95% for videos). In a prospective study (951 patients, 1254 lesions), it demonstrated real-time diagnostic accuracy for the five Correa cascade categories of gastric lesions ranging from 91.71% to 97.13%, with gastric tumor sensitivity (96.70%) significantly exceeding that of specialists (89.01%). It achieves expert-level diagnosis across the entire Correa cascade, enabling endoscopists to better monitor gastric tumors and precancerous lesions. Furthermore, it promotes the application of probe-based confocal laser endoscopy (pCLE) while reducing unnecessary biopsies, providing an efficient and reliable tool for early gastric cancer diagnosis and intervention. These studies indicate that artificial intelligence holds promise as a valuable tool to assist endoscopists, particularly those without specialized pathology expertise, in performing non-invasive diagnosis of gastric cancer and its precancerous lesions via confocal laser endomicroscopy, thereby avoiding invasive histological biopsies.

### 2.4. Multimodal Models

In clinical practice, endoscopy frequently requires the use of multiple imaging modalities. Therefore, compared to AI models built solely on a single modality, integrating two or more modalities into dual-modality or multi-modality AI models holds greater clinical application value. The combination of NBI and magnifying endoscopy enables clear visualization of changes in mucosal microvascular patterns and micro-surface structures, and is widely applied in the diagnosis of gastric cancer and its precancerous lesions [26,27,28]. Yan et al. [29] developed a dual-mode intelligent diagnostic system for automated GIM identification based on the EfficientNetB4 architecture, integrating NBI and ME-NBI images. They retrospectively collected 1880 NBI and ME-NBI endoscopic images from 336 patients. On an independent test set of 80 patients, the system demonstrated diagnostic performance comparable to that of senior endoscopists (sensitivity 91.9% vs. 86.5%, accuracy 88.8% vs. 83.8%), whilst significantly outperforming manual assessment in diagnostic speed (0.05 s per image). Notably, the system also generates attention maps via Grad-CAM to visualize key lesion areas, enhancing model interpretability and demonstrating significant potential for clinical diagnostic support and teaching applications. Hu et al. [30] developed the EGCM model based on the VGG-19 architecture. Trained on 1777 ME-NBI images (295 patients) from three hospitals, it was applied to early gastric cancer (EGC) diagnosis. The model achieved AUC values of 0.808 and 0.813 on internal and external validation cohorts, respectively, demonstrating performance comparable to senior endoscopists (accuracy 0.770 vs. 0.755, *p* = 0.355) and superior to junior practitioners. Endoscopists’ diagnostic capability significantly improved when referencing EGCM. Ueyama et al. [31] developed the AI-assisted CNN-CAD system based on ResNet50, trained and validated using 5574 ME-NBI images (including 3797 EGC cases) and tested on 2300 independent images. It completed analysis of 2300 images in just 60 s, achieving 98.7% accuracy, 98% sensitivity, 100% specificity, and an AUC of 99%. Ikenoyama et al. [32] constructed a convolutional neural network (CNN)-based model for early gastric cancer detection. The training set comprised 13,584 images from WLI, indigo carmine-stained endoscopy (CE) and NBI endoscopy. It was then tested on 2940 images from 140 patients and compared against the diagnostic capabilities of 67 endoscopists (33 certified, 34 non-certified). Results showed the CNN’s diagnostic sensitivity (58.4%) was significantly higher than that of endoscopists (58.4% vs. 31.9%). Notably, the CNN analyzed 2940 images in just 45.5 s, far faster than the 173 min required by endoscopists, offering potential as a real-time tool to alleviate physician workload.

### 2.5. Video and Real-Time Systems

Recently, artificial intelligence technology is progressively evolving from reliance on static images towards integration with dynamic scenarios, providing clinical practice with solutions more closely aligned with practical requirements. Previously, the majority of research developed and tested models using static images, potentially failing to accurately reflect real-world clinical conditions [33,34,35]. Consequently, numerous studies have developed AI models incorporating complex dynamic video footage to deliver more authentic and precise insights in clinical practice.

Ma et al. [36] pioneered a deep learning model based on the YOLOv4 architecture. While initially trained on 3723 magnified optical enhancement images, the model was innovatively optimized and tested using 101 video clips and 117 unedited, full-length videos. Designed to distinguish early gastric cancer, benign lesions, and background mucosa within live video streams, the model achieved 91% sensitivity and 78% specificity on an independent test set. Its overall accuracy (84%) matched that of expert endoscopists, confirming its reliability for dynamic clinical settings.

Similarly, Xu et al. [37] develop the ENDOANGEL system for detecting gastric precancerous lesions using magnifying endoscopy combined with narrow-band imaging (ME-NBI) and blue-light imaging (ME-BLI). Trained on robust data (6250 images and 98 videos), the system achieved diagnostic accuracies of 0.878 and 0.898 for chronic atrophic gastritis (CAG) and gastric intestinal metaplasia (GIM), respectively, during prospective video testing. Its performance significantly outperformed non-experts (CAG: 0.869 vs. 0.750, *p* = 0.008; GIM: 0.888 vs. 0.736, *p* = 0.028). With high positive predictive values (>0.92), this system shows substantial potential to reduce the need for multiple biopsies.

Focusing on conventional white-light imaging (WLI), Wu et al. [38] validated the ENDOANGEL-LD system across 100 video segments and 2010 prospective patients. In video testing, it demonstrated significantly higher sensitivity (100%) and negative predictive value (100%) for gastric tumors compared to an expert panel (85.5% and 86.4%, respectively). Furthermore, Tang et al. [39] developed a real-time DCNN system trained on 35,823 WLI images. Validated using gastroscopy videos from multiple hospitals, the system achieved 88.5% sensitivity at a rapid processing speed of 15 milliseconds per frame. Notably, with AI assistance, the diagnostic sensitivity of trainee endoscopists surged from 50.2% to 94.7%, effectively elevating their capability to expert-level performance.

**Table 1:** Lesion detection and characterization for EGC and precancerous lesions.

Author, Year of Publication	Purpose	Endoscopic Modality	Algorithm	Training Dataset	Test Dataset	Outcome			Compared to Endoscopists
						Accuracy	Sensitivity	Specificity	
Luo, 2019 [13]	Detection of GC	WLI	DeepLab V3+	125,898 images	31,309 images	95.5% (internal validation), 91.5–97.7% (external validation)	94.0% (internal validation), 90.7–98.2% (external validation)	96.1% (internal validation), 91.3–97.9% (external validation)	Comparable to experts; superior to non-experts
Zhang, 2020 [11]	Diagnosis of CAG	WLI	DenseNet	3829 images	1641 images	94.2%	94.5%	94.0%	Superior to experts
Yan, 2020 [29]	Diagnosis of gastric IM	ME, NBI	EfficientNet-B4	1880 images	477 images	88.8%	91.9%	86.0%	Superior to experts
Ikenoyama, 2020 [32]	Detection of EGC	WLI, CE, NBI	SSD	13,584 images	2940 images	NA	58.4%	87.3%	Superior to endoscopists in sensitivity
Tang, 2020 [39]	Real-time detection of EGC	WLI	Darknet-53	35,823 images	10,931 images, 21 videos	85.1–91.2%	85.9–95.5%	81.7–90.3%	Superior to endoscopists
Hu, 2021 [30]	Identification of EGC	ME, NBI	VGG-19	1777 images		77.0%	79.2%	74.5%	Similar to senior endoscopists superior to junior endoscopists
Ueyama, 2021 [31]	Diagnosis of EGC	ME, NBI	ResNet-50	2300 images	5574 images	98.7%	98%	100%	NA
Xu, 2021 [37]	Diagnosis of gastric precancerous conditions	ME-NBI/ BLI	VGG-16	5198 images	98 videos	90.1%, 90.8% (GA, IM internal validation), 89.8%, 96.6% (GA, IM external validation), 87.8%, 89.8% (GA, IM prospective video validation)	89.5%, 88.5% (GA, IM internal validation), 86.4%, 85.9% (GA, IM external validation), 96.7%, 94.6% (GA, IM prospective video validation)	90.7%, 93.0% (GA, IM internal validation), 79.4%, 72.3% (GA, IM external validation), 73%, 83.7% (GA, IM prospective video validation)	Similar to experts and superior to non-experts.
Yuan, 2022 [12]	Diagnosis of gastric lesions	WLI	YOLO	29,809 images	1579 images	85.7%	NA	Not available	Comparable to experts; Superior to non-experts
Tang, 2022 [19]	Diagnosis of EGC	NBI	YOLOv3	13,151 images	1577 images, 20 videos	95.9% (internal validation) 87.6–92.9% (external validation)	98.0% (internal validation), 87.7–96.7% (external validation) 100% (videos)	85.2% (internal validation), 81.1–91.3% (external validation)	Outperforms both junior and senior endoscopists
Ma, 2022 [36]	Real-time differentiation of gastric lesions	M-OE	YOLOv4	3723 images	101 videos, 671 images	84%	91%	78%	Comparable to experts
Wu, 2022 [38]	Real-time diagnosis of gastric neoplasms	WLI	YOLOv3 ResNet-50	9824 images 100 videos		72.0%	100.0%	53.2%	Superior to endoscopists
Dong, 2023 [14]	Diagnosis of early gastric cancer	WLI	DCNN, ResNet-50, YOLOv3 RF	4482 images and 296 videos		86.61% (internal images), 81.10% (internal videos), 77.17% (external images), 88.24% (external videos)	85.22% (internal images), 85.45% (internal videos), 87.30% (external images), 97.06% (external videos)	87.11% (internal images), 77.78% (internal videos), 73.08% (external images), 82.35% (external videos)	Superior to endoscopists
Du, 2023 [40]	Diagnosis of gastric neoplasms in real-time	WLI, WME	CNN	4201 images, 7436 image pairs, and 162 videos		86.54% (images), 90%(videos), 93.55% (prospective test)	89.66% (images) 95.65% (videos) 93.75% (prospective test)	85.33% (images) 88.31% (videos) 93.48% (prospective test)	Superior to endoscopists
Cho, 2024 [24]	Evaluation histopathology of gastric cancer	CLE	EfficientNetV2-S	20,408 images	20,408 images	96.4% (internal validation), 99.0% (external validation)	96.6% (internal validation), 100% (external validation)	96.4% (internal validation), 98.2% (external validation)	Inferior to pathologist
Lei, 2025 [20]	Recognition of EGC	Conversion of WLI to NBI	Stable Diffusion	273 images	11 paired images of WLI-vir-NBI-NBI	81.08% (junior) 84.68% (intermediate), 92.79% (senior)	83.91% (junior), 85.06% (intermediate), 94.25% (senior)	70.83% (junior) 83.33% (intermediate), 87.50% (senior)	Not reported
Liu, 2025 [25]	Diagnosis of Correa cascade of gastric cancer	pCLE	Inception- ResNet V2	41,743 images, 461 videos	11,439 images, 667 videos	91.71–97.13%	98.44% (HGIN/CA), 96.70% (LGIN + HGIN/CA)	97.60% (HGIN/CA) 96.48% (GIM)	Comparable to experts

Abbreviations: NA = not available/not reported in the original study; WLI = white light imaging; NBI = narrow band imaging; CE = chromoendoscopy; EGC = early gastric cancer.

Addressing the need for integrating multiple inputs, Du et al. [40] developed the ENDOANGEL-MM multimodal system, utilizing both white light and weak magnification data from images and videos. The feature-level fusion mode demonstrated optimal performance, significantly exceeding the average accuracy of 13 endoscopists (90.00% vs. 76.17%). It elevated non-specialist accuracy from 70.75% to 85.75% while drastically reducing diagnosis time (30.67 vs. 65.17 min), highlighting the clinical value of dynamic, multimodal AI.

In summary, when evaluating the current landscape of AI for gastric lesion detection, distinct performance differences emerge across modalities and system architectures. AI models based on WLI offer the broadest applicability and highest sensitivity for initial screening. Conversely, NBI/ME-NBI and CLE models demonstrate superior specificity for characterizing microvascular patterns and providing “optical biopsies.” Furthermore, multimodal systems consistently outperform single-modality models by fusing macroscopic context (WLI) with microscopic details (ME), thereby reducing false-positive rates. Most importantly, while preliminary static-image models established foundational accuracy in controlled settings, the strongest and most clinically relevant evidence today derives from prospective, multicenter video-based trials (such as the ENDOANGEL studies). These real-time systems, capable of processing live video streams without latency, represent the true maturity of AI readiness for routine clinical implementation.

## 3. Deep Learning-Assisted Margin Delineation for Gastric Cancer and Precancerous Lesions

Endoscopy combined with histopathological examination constitutes the gold standard for diagnosing gastric cancer and precancerous lesions. Ensuring biopsy sampling from the most representative areas of the lesion is crucial for accurate diagnosis and preventing missed diagnosis caused by “sampling bias”. Therefore, the precise delineation of lesion margins is paramount, as it provides accurate guidance for biopsy placement and is critical for detecting early gastric cancer. Therapeutically, numerous guidelines recommend endoscopic resection (ER) as the primary treatment for early gastric cancer. Compared to conventional surgery, ER is significantly more cost-effective and less invasive. However, its applicability is highly dependent on a precise preoperative assessment. The extent of mucosal resection correlates closely with the incidence of complications such as perforation, delayed stricture, and hemorrhage [41,42,43]. Furthermore, inaccurate assessment of early gastric cancer (EGC) margins may lead to incomplete resection or positive endoscopic resection margins, necessitating additional surgery or increasing five-year mortality rates [44]. Consequently, precise preoperative delineation of lesion margins is crucial for treatment selection and ensuring the thoroughness of endoscopic resection.

In Japan, chromoendoscopy (CE) using indigo carmine solution following routine white-light endoscopy (WLE) is the standard method for delineating gastric tumor margins [45]. However, diagnostic inaccuracies persist in approximately 20% of EGC patients [46]. An et al. [47] developed an artificial intelligence model named ENDOANGEL based on a fully convolutional network. This model utilized 1244 images from 536 patients (including indigo carmine-stained endoscopy and white-light endoscopy images) to accurately delineate resection margins of early gastric cancer under both stained and white-light endoscopy. The researchers evaluated the model using 355 internal test images and 10 endoscopic submucosal dissection (ESD) procedure videos. Results demonstrated that ENDOANGEL achieved delineation accuracy comparable to expert manual annotations in the image test set (85.7% for indocyanine green endoscopy, 88.9% for white light endoscopy), while its predictions in the video test fully covered all high-grade intraepithelial neoplasias and cancerous regions. Notably, the model processes images extremely rapidly at 80 milliseconds per frame, demonstrating potential for real-time endoscopic surgical assistance.

Compared to WLI, NBI is more accurate for delineating lesion boundaries [48]. Hong et al. [49] developed a deep learning-based artificial intelligence model to delineate the margins of early gastric cancer on narrow-band imaging (NBI) and near-focus narrow-band imaging (NF-NBI) endoscopic images. Trained on 2861 images, the model was evaluated against endoscopists of varying experience levels on a test cohort. Results demonstrated high concordance between the model’s delineated lesion margins and those of senior endoscopists, with significantly superior performance compared to junior and intermediate practitioners. This indicates substantial potential for the AI model in assisting endoscopists to achieve precise resection of early gastric cancer and reduce the risk of secondary surgery. Okumura et al. [50] developed a novel computer-aided diagnosis system based on unsupervised machine learning to automatically delineate early gastric cancer margins within magnified narrow-band imaging endoscopy images. Employing an innovative approach combining superpixel segmentation and k-means clustering, the system generates multiple margin candidates without requiring pre-annotated data. On a test set comprising 23 ME-NBI images, the system generated boundaries highly similar to those manually delineated by senior endoscopists, with mean Euclidean distances below 10 pixels across multiple cases. It effectively outlined complex lesion borders consistently with expert judgements, offering potential to assist less experienced clinicians and provide valuable reference for all endoscopists_._ Ling et al. [51] proposed an artificial intelligence system for delineating early gastric cancer margins under magnified narrow-band imaging (ME-NBI) endoscopy. It was trained and tested on 928 images from 132 patients and 742 images from 87 patients, respectively. The model achieved high-precision boundary delineation, with delineation accuracy rates of 82.7% and 88.1% for differentiated and undifferentiated early gastric cancers, respectively, under an overlap ratio criterion of 0.80.In unprocessed real-time endoscopic video, the system achieved real-time boundary delineation at 25 frames per second, automatically and accurately mapping the cancerous region when the physician froze the image [51] (Table 2).

These AI margin delineation tools can be well integrated into the standard workflow of endoscopic submucosal dissection (ESD). Before surgery, AI helps physicians complete accurate lesion mapping and formulate surgical plans. During ESD, the system provides real-time intraoperative guidance to ensure complete lesion resection. After the operation, it can also be used for postoperative quality review to standardize endoscopic treatment quality.

## 4. Invasion Depth Prediction

Tumor invasion depth is a critical factor in determining gastric cancer treatment decisions. For mucosal carcinoma (M) and cancers with submucosal invasion <500 μm (SM1), endoscopic submucosal dissection (ESD) often achieves curative resection; whereas deeply invasive gastric cancers require surgical resection [61]. Studies indicate that conventional endoscopy achieves only 73.7% accuracy in predicting tumor depth in early gastric cancer (EGC) [62]. This has spurred the application of deep learning (DL) in predicting gastric cancer depth and identifying suitable candidates for endoscopic resection using endoscopic images.

The accuracy of invasion depth prediction is intrinsically linked to clinical decision-making. Overestimation of the invasion depth (e.g., misclassifying M/SM1 as SM2) may lead to unnecessary radical gastrectomy, exposing the patient to higher surgical morbidity and diminished quality of life. Conversely, underestimation (misclassifying SM2+ as mucosal lesions) can result in incomplete endoscopic resection, leaving residual malignant tissue and necessitating secondary salvage surgeries. Therefore, the prospective evaluation of AI systems in real-world treatment-planning contexts is urgently needed to confirm their safety and reliability.

Uema et al. [60] pioneered an artificial intelligence model based on convolutional neural networks (CNNs) to distinguish mucosal or superficial submucosal invasion (M-SM1) from deep submucosal invasion (SM2 or deeper) in early gastric cancer using endoscopic ultrasound (EUS) images. Their dataset comprised 8280 EUS images from 559 cases. In an internal validation cohort, diagnostic accuracy (82.2%) matched that of endoscopy specialists (81.9%) and significantly outperformed non-specialists (68.3%). In an external multicenter validation cohort, diagnostic accuracy (74.1%) showed no significant difference from real-time diagnoses by institution-based specialists (75.5%), marking the first demonstration that AI can achieve expert-level performance in diagnosing early gastric cancer infiltration depth via EUS. Zhu et al. [52] constructed a ResNet50-based convolutional neural network computer-aided detection system specifically designed to distinguish P0 (M/SM1) from P1 (SM2 and above) gastric cancer invasion depth under conventional white-light endoscopy. The dataset comprised 790 training images and 203 test images. The model processed rapidly, evaluating all 203 test images in just 36 s, enabling immediate post-endoscopy determination of lesion invasion depth with high accuracy (89.16%) and specificity (95.56%). Notably, both accuracy and specificity significantly surpassed those of 17 endoscopists (including 8 experienced practitioners), effectively distinguishing EGC from tumors with deep submucosal invasion while minimizing the risk of overestimating invasion depth, thereby reducing unnecessary gastrectomy procedures). Nagao et al. [55] developed three independent artificial intelligence systems based on ResNet50, utilizing endoscopic images from white light imaging, non-magnifying narrow band imaging, and indigo carmine contrast imaging, respectively, to predict gastric cancer invasion depth. Their task was to distinguish M/SM1 lesions from SM2 or deeper lesions. The dataset comprised 16,557 images from 1084 lesions. The white-light AI model achieved the highest lesion-level accuracy at 94.5%, with a sensitivity of 84.4% and remarkable specificity of 99.4%. AI systems for all three imaging modalities (WLI, NBI, Indigo) demonstrated excellent performance with no significant differences in accuracy. Goto et al. [59] developed an AI model based on EfficientNetB1 to distinguish mucosal-layer carcinoma (M) from submucosal-layer carcinoma (SM) in early gastric cancer under white-light imaging. Trained on 500 images and validated on 200 external test images, the model achieved 72.5% diagnostic accuracy, outperforming endoscopists at 70.0%. Through collaborative diagnosis between AI and endoscopists, accuracy can be further enhanced to 78.0%.

Compared to static images, incorporating dynamic video during model development and validation better reflects real clinical scenarios. Kim et al. [57] developed an artificial intelligence model (video classifier, VC) based on the VGG-16 architecture for real-time prediction of the depth of invasion in early gastric cancer. Trained on 354 white-light endoscopy videos of EGC, the model employs a sliding window approach, processing seven consecutive frames per sequence to learn subtle dynamic changes. When tested on 67 independent videos, the VC demonstrated high sensitivity (82.3%), specificity (85.8%), and accuracy (83.7%). A comparison with a static image-based model (IC v2) revealed a significantly higher sensitivity (82.3% vs. 33.6%). Furthermore, VC exhibited a lower standard deviation in output probabilities (0.096 vs. 0.289), indicating greater prediction consistency. This demonstrates that AI models trained on video data can more accurately and reliably simulate real-world endoscopic environments, offering the potential to assist clinicians in decision-making. Wu et al. [58] developed a deep learning system named ENDOANGEL to assist in the diagnosis of early gastric cancer within real-time video streams, simultaneously achieving lesion detection, early gastric cancer identification, depth of invasion prediction, and differentiation status assessment. The system was tested using data from 100 endoscopic videos (including 37 cases of early gastric cancer) and underwent real-time human–machine comparisons with 46 endoscopists from multiple hospitals nationwide. Results demonstrated that the system’s sensitivity in identifying early gastric cancer (100%) significantly exceeded that of endoscopists (87.13%) and matched clinician performance in predicting depth of invasion (78.57% accuracy) and differentiation status (71.43% accuracy). Crucially, in simulations mirroring real clinical workflows, its overall diagnostic accuracy reached 96%, surpassing endoscopists across all proficiency levels and highlighting its substantial potential as a clinical aid.

## 5. Blind Spots Monitoring

Endoscopic examination of the entire stomach is a crucial prerequisite for diagnosing gastric cancer and its precancerous lesions. To avoid overlooking blind spots, standardized operating procedures and guidelines have been established for endoscopic examinations. The European Society of Gastrointestinal Endoscopy guidelines recommend capturing at least ten images of the stomach’s core anatomical locations. Japan, however, mandates a minimum of twenty-two photographs under its Systematic Screening Protocol for the Stomach (SSS), ensuring comprehensive coverage of all critical gastric regions to eliminate blind spots [63,64]. However, challenges persist, including endoscopists potentially overlooking certain gastric areas due to subjective judgment or insufficient operational experience, coupled with low compliance in community settings and inadequate oversight. These factors hinder strict adherence to protocols, directly compromising endoscopic quality [65,66,67,68]. Advances in artificial intelligence have spurred research into its application for blind spot monitoring. Wu et al. [53] employed a deep convolutional neural network (DCNN) system to detect gastric cancer and identify blind spots. Training utilized two datasets derived from white light, narrow-band imaging (NBI), and blue light imaging (BLI) endoscopic images: one comprising 3170 early gastric cancer images and 6541 benign images, and another containing 24,549 images of different gastric regions. A gastric grid model was developed to generate a virtual stomach model, achieving 92.5% accuracy in distinguishing early gastric cancer from non-malignant lesions, with a sensitivity of 94.0% and specificity of 91.0%, outperforming endoscopists of all proficiency levels. The system accurately segmented EGD images into 10 regions with 90.0% accuracy and into 26 regions with 65.9% accuracy, matching expert performance. Notably, when applied to unprocessed gastroscopy videos, the DCNN system accurately displays regions covered synchronously with the EGD procedure, actively tracking suspicious neoplastic lesions without blind spots. This alleviates pressure and workload for endoscopists during real-time gastroscopy examinations. In another study, Wu et al. [54] developed the WISENSE model based on deep convolutional neural networks and deep reinforcement learning methods, enabling real-time blind spot quality monitoring and control. Trained on 34,513 white-light endoscopic images verified by at least four endoscopists and validated across 107 real endoscopic videos, WISENSE achieved a 90.0% accuracy rate in blind spot detection. Interestingly, in our center’s randomized controlled trial, the blind spot rate (defined as the proportion of 26 observation sites missed) was 5.9% in the WISENSE group, significantly lower than the 22.5% in the control group (without AI) (*p* < 0.001). This demonstrates that artificial intelligence can effectively reduce blind spots and improve the overall quality of upper gastrointestinal endoscopy. Li et al. [56] developed the AI-based IDEA system, training it on 170,297 white-light endoscopic images and 5779 endoscopic video segments. The testing phase utilized 3100 images and 129 endoscopic videos, where the system achieved remarkable specificity and accuracy of 99.91% and 99.83%, respectively, in image testing, while achieving 93.32% specificity and 95.30% accuracy for video testing. Both demonstrated high accuracy in real-time gastric location identification. Building upon this, Li et al. [69] conducted a large-scale multicenter validation of IDEA, incorporating 17,787 patients from 12 hospitals. IDEA performed blind monitoring, quality scoring, and procedural duration recording for all gastroscopy videos. Results confirmed that gastroscopy quality (measured by IDEA total score) showed significant positive correlations with both gastric cancer and early gastric cancer detection rates, and this correlation could be monitored via IDEA.

## 6. Limitations and Future Prospects

While AI-assisted endoscopy has achieved diagnostic performance rivaling that of specialist endoscopists, several critical limitations must be addressed before its widespread clinical implementation. These challenges can be categorized into data, technical, clinical, and regulatory domains.

### 6.1. Data-Related Limitations

Currently, the datasets used for AI model development remain highly limited. Most studies rely on retrospective, single-center data comprising predominantly high-quality images, which fails to represent the noise and artifacts of real-world clinical scenarios [11,12,29,32,36,49,55,60]. Furthermore, existing datasets suffer from spectrum bias, focusing heavily on early gastric cancer while neglecting other lesions like inflammation, intestinal metaplasia, and polyps. To overcome this, future research must establish standardized, multi-center data repositories. Techniques such as federated learning should be incorporated to expand dataset diversity while mitigating data bias and protecting patient privacy.

### 6.2. Model and Technical Limitations

Model generalizability remains a major technical hurdle. Algorithms trained on images from a single endoscopy brand frequently experience performance drops when applied to diverse equipment across different centers [20,32,39,56,58]. Additionally, most existing systems are single-task (e.g., detection only) and lack an integrated pipeline encompassing characterization, margin delineation, and invasion depth. To address these complex, multi-task clinical requirements, the next generation of AI-assisted endoscopy will likely be driven by Foundation Models, Vision-Language Models (VLMs), and Large Language Models (LLMs). These pre-trained, large-scale models offer unprecedented zero-shot generalization capabilities and can integrate visual findings with patient histories to generate comprehensive, automated diagnostic workflows.

### 6.3. Clinical Implementation and Explainability

The inherent “black box” nature of deep learning algorithms poses a significant barrier to clinical acceptance. Clinicians often hesitate to trust AI systems when the underlying decision-making logic is opaque. To tackle this trust issue, adopting an “Explainable AI (XAI)” architecture must become the industry standard. Future models should generate interpretability reports (e.g., heatmaps combined with textual rationales) detailing the corresponding clinical features used for predictions. This transparency will enable endoscopists to fully comprehend and validate the algorithmic output.

### 6.4. Regulatory and Ethical Considerations

Finally, the widespread adoption of AI technologies raises critical societal and regulatory concerns. While AI systems for colorectal polyp detection have already been granted Class III medical device certifications and proven effective in real-world settings [70,71,72,73,74,75,76], gastric AI models urgently require similarly robust prospective validation. Ethically, AI must be strictly positioned as a diagnostic adjunct rather than a replacement, ensuring that overreliance does not hinder the professional development and skill acquisition of junior practitioners. Moving forward, comprehensive regulatory frameworks must be established to address patient safety, data privacy, legal liability, and long-term cost-effectiveness.

A concise summary outlining the current clinical readiness, maturity levels, and primary limitations of major AI applications in gastric endoscopy is provided in Table 3.

## 7. Conclusions

AI-assisted endoscopy achieves diagnostic performance comparable to or even better than specialist endoscopists in lesion detection, margin delineation, invasion depth assessment and blind spot monitoring, and it can effectively improve diagnostic ability for junior endoscopists and primary medical institutions. Current AI systems still have limitations in data quality, model generalizability and interpretability. AI can only serve as an auxiliary tool for clinicians; large-scale prospective validation and standardized management are required before widespread clinical application.

## Figures and Tables

**Table 2:** Margin delineation, prediction of invasion depth and blind spot monitoring for gastric cancer and precancerous lesions.

Author, Year of Publication	Purpose	Endoscopic Modality	Algorithm	Training Dataset	Test Dataset	Outcome			Compared to Endoscopists
						Accuracy	Sensitivity	Specificity	
Zhu, 2019 [52]	Diagnosis of the invasion depth of gastric cancer	WLI	ResNet50	790 images	203 images	89.16%	76.47%	95.56%	Superior to endoscopists
Wu, 2019 [53]	Detection of EGC without blind spots	WLI	VGG-16 ResNet-50 TensorFlow	9152 images	NA	92.5%	94%	95%	Superior to endoscopists
Wu, 2019 [54]	Real-time monitoring of blind spots	WLI	DCNN DRL	NA	107 videos	90.02%	87.57%	95.02%	Superior to endoscopists
An, 2020 [47]	Delineation of EGC resection margin	CE, WLE	VGG-16	889 images	355 images, 10 videos	85.7% (CE images), 88.9% (WLE images), 100% (ESD videos)	81.7% (images), 89.5% (videos)	NA	Comparable to experts
Nagao, 2020 [55]	Prediction of the invasion depth of gastric cancer	WLI, NBI, Indigo	ResNet-50	16,557 images		94.5% (WLI) 94.3% (NBI) 95.5% (Indigo)	84.4% (WLI) 75% (NBI) 87.5% (Indigo)	99.4% (WLI) 100% (NBI) 100% (Indigo)	NA
Ling, 2021 [51]	Delineation of the margins of EGC	ME-NBI	UNet++	928 images	742 images	82.7% (Undifferentiated EGC), 88.1% (Undifferentiated Early-Stage Gastric Cancer)	NA	NA	NA
Li, 2021 [56]	Monitoring blind spots in real-time	WLI	SqueezeNet Inception- V3	170,297 images 5779 videos		99.83% (images) 95.30% (videos)	99.83% (images), 95.30% (videos)	99.83% (images), 95.30% (videos)	Comparable to endoscopists
Okumura, 2022 [50]	Determination of an accurate demarcation line (DL) between the cancerous lesions and background mucosa	ME-NBI	NA	NA	23 images 11 cases	NA	NA	NA	Comparable to experts
Kim, 2022 [57]	Real-time prediction of invasion depth in EGC	WLI	VGG-16	354 videos	67 videos	83.7%	82.3%	85.8%	Not reported
Wu, 2022 [58]	Prediction of EGC and its Invasion Depth	WLI ME-NBI	YOLOv3 ResNet-50 TensorFlow	3407 images	228 images 100 videos	88.2%	90.5%	85.3%	Comparable to endoscopists
Goto, 2023 [59]	Diagnosis of the invasion depth of EGC	WLI	EfficientNetB1	500 images	250 images	77%	76%	78%	Superior to endoscopists in sensitivity
Uema, 2024 [60]	Diagnosis of the invasion depth of EGC	EUS	ResNet-34 CycleGAN EfficientNetV2-L	3451 images	1726 (internal validation images), 3103 (external validation images)	82.2% (internal validation), 74.1% (external validation)	63.4% (internal validation), 73.1% (external validation)	90.4% internal validation), 75.0% (external validation)	Superior to non-expert and comparable to experts (internal validation), comparable to experts (external validation)
Hong, 2025 [49]	Boundary recognition of EGC	NBI, NF-NBI	TGANet LDNet	980 (NBI images) 1273 (NF-NBI images)	235 (NBI images) 373 (NF-NBI images)	72.77% (Mode1), 91.74% (Model2), 97.73%, (Model 3)	NA	NA	Comparable to senior endoscopists

Abbreviations: NA = not available/not reported in the original study; WLI = white light imaging; NBI = narrow band imaging; CE = chromoendoscopy; EGC = early gastric cancer.

**Table 3:** Summary of current maturity, clinical readiness, and limitations of major AI applications in gastric.

AI Application Area	Current Maturity Level and Clinical Readiness	Primary Limitations	Future Directions
Lesion Detection and Characterization	High (Several systems commercialized and approved as medical devices; widely tested in real-time)	Overfitting to single-center data; false positives in severe inflammation or atypical lesions.	Multi-center federated learning; integration with generative AI for robust feature extraction.
Blind Spot Monitoring	Moderate to High (Demonstrated efficacy in real-time video; improving quality control scores)	High computational demand; workflow disruption if false alerts are frequent; relies on strict protocol compliance.	Development of lightweight algorithms for edge computing without latency.
Margin Delineation	Moderate (Primarily in research/validation phase; shows promise for assisting ESD/EMR)	Highly dependent on image quality and specific imaging modalities; requires pixel-level annotation for training.	Zero-shot segmentation using Medical Foundation Models; real-time dynamic overlay in surgical monitors.
Invasion Depth Prediction	Low to Moderate (Mostly retrospective studies; limited prospective real-time application)	Struggle to differentiate submucosal micro-invasion precisely; heavily relies on endoscopist’s optical maneuvering.	Multi-modal integration combining WLI/NBI with Endoscopic Ultrasonography (EUS) and clinical biomarkers.

## Data Availability

No new data were created or analyzed in this study. Data sharing is not applicable to this article.
